# Metformin targets the GTPase Rac1 to inhibit prostate cancer cell migration

**DOI:** 10.1186/2049-3002-2-S1-O24

**Published:** 2014-05-28

**Authors:** Béatrice Dirat, Isabelle Ader, Muriel Golzio, Amel Mettouchi, Kathiane Laurent, Frédéric Larbret, Bernard Malavaud, Mireille Cormont, Emmanuel Lemichez, Jean François Tanti, Frédéric Bost

**Affiliations:** 1 INSERM U1065, C3M, Nice, France; 2 CNRS U5089, IPBS, Toulouse, France; 3 Rangeuil Hospital, Toulouse, France

## Background

The anti-diabetic drug metformin has been shown to affect cancer cell metabolism [1,2] and to display anti-tumoral properties in numerous cancers [3,4], however, its role in the formation of metastases remains poorly documented. Cell migration is a critical step in the progression of prostate cancer to the metastatic state, the lethal form of the disease.

## Results

We show here that metformin reduces the occurrence of metastases in an orthotopic metastatic prostate cancer cell model established in nude mice. As predicted, metformin hampers cell motility in PC3 and DU145 prostate cancer cells and triggers a radical reorganization of the cell cytoskeleton. The small GTPase Rac1 is a master regulator of cytoskeleton organization and cell migration. We report that metformin inhibits Rac1 GTPase activity by interfering with some of its multiple upstream signaling pathways, namely P-Rex1 (a Guanine nucleotide exchange factor and activator of Rac1), cyclic AMP and CXCL12/CXCR4, resulting in decreased migration of prostate cancer cells. Importantly, overexpression of a constitutively active form of Rac1 (Rac1-Q61L or Rac1-V12), or P-Rex1 as well as the inhibition of the adenylate cyclase were able to reverse the anti-migratory effects of metformin.

## Conclusion

Our results establish a novel mechanism of action for metformin through Rac1 and highlight its potential anti-metastatic properties in prostate cancer.

## References

[B1] El-Mir MY Nogueira V Fontaine E Averet N Rigoulet M Leverve X Dimethylbiguanide inhibits cell respiration via an indirect effect targeted on the respiratory chain complex I J Biol Chem 2000 275 223 8 10.1074/jbc.275.1.223 10617608

[B2] Ben Sahra I Laurent K Giuliano S Larbret F Ponzio G Gounon P Targeting cancer cell metabolism: the combination of metformin and 2-deoxyglucose induces p53-dependent apoptosis in prostate cancer cells Cancer Res 2010 70 2465 75 10.1158/0008-5472.CAN-09-2782 20215500

[B3] Ben Sahra I Laurent K Loubat A Giorgetti-Peraldi S Colosetti P Auberger P The antidiabetic drug metformin exerts an antitumoral effect in vitro and in vivo through a decrease of cyclin D1 level Oncogene 2008 27 3576 86 10.1038/sj.onc.1211024 18212742

[B4] Zakikhani M Dowling R Fantus IG Sonenberg N Pollak M Metformin is an AMP kinase-dependent growth inhibitor for breast cancer cells Cancer Res 2006 66 10269 73 10.1158/0008-5472.CAN-06-1500 17062558

